# Alcohol and marijuana use while driving--an unexpected crash risk in Pakistani commercial drivers: a cross-sectional survey

**DOI:** 10.1186/1471-2458-12-145

**Published:** 2012-02-27

**Authors:** Mohammed Umer Mir, Imran Khan, Bilal Ahmed, Junaid Abdul Razzak

**Affiliations:** 1 Department of Emergency Medicine, Aga Khan University Hospital, First floor Stadium Road, Karachi, Pakistan; 2 King Edward Medical University, Lahore, Pakistan; 3 Department of Medicine, Aga Khan University Hospital, Karachi, Pakistan; 4 Department of Emergency Medicine, Aga Khan University Hospital, Karachi, Pakistan

## Abstract

**Background:**

A significant proportion of road traffic crashes are attributable to alcohol and marijuana use while driving globally. Sale and use of both substances is illegal in Pakistan and is not considered a threat for road traffic injuries. However literature hints that this may not be the case. We did this study to assess usage of alcohol and marijuana in Pakistani commercial drivers.

**Methods:**

A sample of 857 commercial bus and truck drivers was interviewed in October 2008 at the largest commercial vehicle station in Rawalpindi and Islamabad, Pakistan. Time location cluster sampling was used to select the subjects and a structured questionnaire was used to assess the basic demographic profile, substance abuse habits of the drivers while on the road, and reasons for usage of illicit substances while driving were recorded. Self reported information was collected after obtaining informed consent. Chi square and fisher exact tests were used to assess differences between groups and logistic regression was used to identify significant associations between driver characteristics and alcohol and marijuana use.

**Results:**

Almost 10% of truck drivers use alcohol while driving on Pakistani roads. Marijuana use is almost 30% in some groups. Statistically different patterns of usage are seen between population subgroups based on age, ethnicity, education, and marital status. Regression analysis shows association of alcohol and marijuana use with road rage and error behaviours, and also with an increased risk of being involved in road crashes. The reported reasons for using alcohol or marijuana show a general lack of awareness of the hazardous nature of this practice among the commercial driver population.

**Conclusion:**

Alcohol and marijuana use is highly prevalent in Pakistani commercial drivers. The issue needs to be recognized by concerned authorities and methods such as random breath tests and sobriety check points need to be employed for proper law enforcement.

## Background

Road traffic crashes (RTCs) account for more than 1.2 million lives lost annually across the globe [1]. This loss is accompanied by almost 50 million injuries, an important contributor to the global disability burden [2]. Most of this burden is borne by the low and middle income countries of the world [1]. By the year 2030, road traffic injuries (RTIs) will be the fifth leading cause of death [2], most of this increase is projected to be in the low-middle income countries (LMICs).

Intoxicants such as alcohol and marijuana affect the mental state of drivers leading to altered perceptions and delayed reactions, increasing the risk for having RTCs [3-6]. Alcohol results in impairment of brake reaction time, speed control, steering responsiveness and lane control, while marijuana causes deficits in tracking, attention, reaction time, short-term memory, hand eye coordination, decision making, and concentration [7,8]. Alcohol also increases the tendency of involvement in high risk behaviors on the road such as speeding [9,10]; crash culpability is directly associated with intoxicant use [11]. While up to twenty-one percent of road traffic crashes may be attributable to alcohol consumption alone in some regions of the world [12], alcohol consumed in combination with marijuana compounds the risk of an RTI [13].

Commercial heavy vehicle crashes due to substance abuse can be very hazardous for both the drivers and for other vehicle occupants involved in the crash [14]. This is kept in check in developed countries by strict substance abuse monitoring mechanisms for this population leading to low levels of usage [15,16]. However studies from low-middle income countries still show 4%-69% of injured drivers having alcohol in their blood [17]; although alcohol use is not well documented from the developing world [18]. A significant proportion of commercial drivers use stimulants to keep awake and relieve fatigue during their long work schedules. This usage is also associated with increased risk for crashes in this population [14,19-21].

Pakistan, a low income country, has the fifth highest annual number of road traffic injury related deaths (40,000) in the world [2]. Commercial drivers contribute to 60% of this burden [2]. Although laws against *driving while intoxicated *(DWI) do exist in Pakistan [2] and use of alcohol is completely prohibited for all drivers, the effectiveness of these laws in controlling the problem is questionable [2,22]. As an Islamic state, the sale and use of alcohol and marijuana is banned for the general public, and they are not considered potential contributors to road traffic crashes [2,23,24]. But with changing patterns of alcohol usage [25], with the labor class having the highest prevalence of alcohol use [26], and with marijuana being a common illicit drug of abuse here [27], Pakistani commercial drivers may not be immune to this driving hazard. This study was designed to assess the magnitude of alcohol and marijuana usage by commercial drivers on Pakistani roads.

## Methods

### Study design

We conducted a cross sectional survey in October 2008 at the Pir Wadhai Bus/Truck station, Rawalpindi, which is a major segregation site of commercial drivers and vehicles in Pakistan. The study site lies on the major route of goods and passenger transport (Karachi to Khyber), with drivers driving across Pakistan stopping here for loading or offloading goods for transportation to other cities/provinces, or for passenger transport. Subjects included in the study were those individuals who either transported goods in heavy vehicles (trucks), or drove vehicles carrying more than 15 passengers across the country. All drivers driving a commercial vehicle, part time or full time, and who drove more than 160 km daily were eligible to be included in the study. Drivers driving on intra city routes and those who had a language barrier problem were excluded.

A structured questionnaire was designed and translated into Urdu, the local language, and self reported information was collected, through interviews. We developed the questionnaire with the help of a panel of experts on road traffic injuries in Pakistan. The questions were then discussed with a group of professional drivers following which pre-testing was done on 90 commercial drivers (10% of the sample size) by data collectors under the supervision of the principal investigator. Some questions had to be rephrased and the sequence of questions was reorganized based on the feedback from the discussion and pre-testing. We decided to ask the questions on personal marijuana or alcohol use at the end of the interview in order to minimize non-response. Interviews were done privately where participants could not be overheard and they were assured of confidentiality of the divulged information. Drivers were asked if they used alcohol or marijuana, while driving, and if yes, then how frequently. Their responses were categorized, ranging from "always" to "never". The drivers' perceptions about risks associated with substance abuse and driving were also assessed. They were asked about their opinions on why commercial drivers resort to alcohol and marijuana use while they drive (1. "Why do you think commercial drivers drink alcohol while driving?; 2. Why do you think commercial drivers use marijuana while driving?"). These opinions were recorded as open responses, in the drivers' own words, and were later on coded at the time of editing. This information was taken prior to asking about personal substance abuse and was collected from all drivers. In addition to alcohol and marijuana use, they were asked about stimulant pill usage and frequency while driving. Also, basic socio demographic information such on age, sex, ethnicity, education, income and marital status was collected from the drivers.

Time location cluster sampling [28] was used as the sampling technique. A cluster was defined as a "stand" at which vehicles were parked for transport of goods or passengers at any one of the three 8 hr sampling time intervals. In all, 59 physical sites (stands) were present at the bus station, which were converted to a total of 177 time location clusters. We randomly selected 78 clusters and recruited the study participants from each one of these. Sampling was done at three eight hour time intervals during the day so as to get a representative sample of drivers driving at different times of the day. Finally, eleven subjects were recruited from each cluster after obtaining informed consent.

### Sample size

During our literature review, we did not come up with any estimates for the proportion of commercial or general drivers using alcohol or marijuana while driving for Pakistan. Thus we calculated the sample size required for the study using a proportion of 0.5 which gives the maximum sample size for estimation of any prevalence. Using a 95% confidence level, a bound on error of 0.05 and a design effect of 2, the minimum required sample size for the study objectives was 769. We inflated the required sample size by 10% to cater for the non response expected in the study. The final sample size required was of 846 subjects and 857 subjects were recruited for the study.

### Statistical analysis

The probability of selection of the subjects in the different clusters was not proportional to size and a fixed number of individuals were taken from each cluster, 11, in order to achieve the required sample size. We performed weighted analysis to overcome this issue. Weights were calculated based on the size of the cluster, assessed by the average number of vehicles parked at that cluster. Basic descriptive statistics were calculated for estimation of prevalence of the different characteristics in these commercial drivers. Estimates were calculated for the overall population, and separately for truckers and passenger vehicle drivers. Differences in the estimates of the two groups were assessed by using student's t test, chi square test, and fisher-exact test where appropriate. Logistic regression was used to calculate the unadjusted ORs for association with having a history of a crash in the last five years. Data analysis was done using the Statistical Package for the Social Sciences (SPSS) version 17 with the complex sample analysis module.

### Ethical approval

Ethical approval was obtained for the study from the *Ethics Review Committee *of the Aga Khan University, Karachi.

## Results

A sample of 857 commercial drivers was interviewed with a total response rate of 81%. All the individuals included in the sample were male, no female was found in this profession. There were two major categories of drivers, those driving load carrying vehicles and those involved in the transport of passengers. Age ranged from 18 yrs to 68 yrs and the mean age for all drivers was found to be 38.3 (95% CI 37.6-38.9). Most of these drivers belonged to the Punjabi ethnicity (45%). The mean of number of completed school years was 5.2 (95% CI 4.9-5.4) in the total but was 4.6 (95% CI 4.4-4.9) in the truckers compared to 5.8 (95% CI 5.5-6.1) in the passenger vehicle drivers, which is significantly higher

Alcohol was used by more than 6% of the sample (Table 1). The rate was significantly higher in truck drivers and showed an increasing trend with age (Table 2). People above 50 yrs of age had a usage rate of 11.3%. Drivers of Sindhi ethnicity exhibited the highest prevalence of DWI (15%) followed by Pathans (8.2%). No statistically significant association was present between years of education and alcohol use, although the rate of usage was highest in drivers with more than 10 years of schooling (13%). Alcohol usage did not vary much across different income groups. Drivers having had a crash in the last five years had an alcohol usage rate of more than 12% compared to only 5.5% in drivers who had not had an accident.

**Table 1:** Prevalence of substance abuse while driving among commercial drivers

	Use of stimulant pill to remain awake while driving n(%)	Use of marijuana while driving n(%)	Use of alcohol while driving n(%)
**Never**	791 (92.3)	661 (77.1)	803 (93.8)

**Occasionally**	44 (5.1)	81 (9.4)	38 (4.4)

**Quite often**	10 (1.2)	89 (10.4)	10 (1.1)

**Always**	12 (1.3)	26 (3)	6 (0.7)

**Total Users**	66 (7.7 ± 1.8)	196 (22.8 ± 2.8)	54 (6.3 ± 1.6)

**Table 2:** Prevalence of alcohol and marijuana use among commercial drivers (sub categories) of Pakistan

	Participants n(%) (N = 857)	Alcohol users (n = 54) (n(%, 95% CI))	Marijuana Users (n = 196) (n (%, 95%CI))	Alcohol & Marijuana (n = 39) (n (%, 95%CI))
**Type of respondent**				

Truckers	461 (53.8)	46 (9.9, 6.8-14.1)	137 (29.9, 24.9-35.4)	35 (7.7, 5-11.8)

Passenger Vehicle Drivers	396 (46.2)	8 (1.9, 1.2-3.2)	59 (14.7, 10.9-19.5)	4 (0.9, 0.5-1.8)

*p-value*		< 0.001	< 0.001	< 0.001

**Age (yrs)**				

18-29	217 (25.3)	9 (4.3, 2.5-7.5)	47 (21.7, 16.9-27.3)	8 (3.7, 2-6.9)

30-39	303 (35.4)	16 (5.3, 3.4-8.3)	76 (25.1, 20.3-30.5)	12 (4.1, 2.4-7.1)

40-49	232 (27.1)	17 (6.8, 4.6-9.8)	51 (21.8, 17.4-26.9)	8 (3.5, 1.9-6.5)

≥ 50	105 (12.3)	12 (11.3, 6.2-19.7)	22 (21.4, 15.5-28.7)	11 (10.1, 5.8-17.1)

*p-value*		0.03	0.56	0.006

**Ethnicity**				

Punjabi	385 (44.9)	15 (3.5, 2.3-5.3)	65 (17, 13.6-21)	8 (2, 1.1-3.5)

Sindhi	62 (7.2)	9 (15.1, 8.6-25.3)	32 (51.8, 41.8-61.7)	9 (15.1, 8.6-25.3)

Pathan	242 (28.2)	20 (8.2, 4.9-13.6)	51 (21.1, 16.4-26.7)	16 (6.6, 3.4-12.2)

Kashmiri	102 (11.9)	5 (5.2, 1.6-8.7)	33 (31.8, 24.1-43.2)	3 (3.1, 1.3-7.4)

Others	66 (7.7)	5 (7.8, 0.7-9.6)	15 (22.7, 15.5-31.8)	3 (4.8, 2.1-10.8)

*p-value*		0.002	< 0.001	< 0.001

**Education (yrs of schooling)**				

No schooling	130 (15.2)	8 (6.2, 3.1-12.2)	28 (21.7, 15.4-29.7)	5 (3.8, 1.3-10.4)

1-5 yrs	332 (38.7)	26 (7.9, 5.1-12.1)	89 (26.7, 21.8-32.2)	23 (6.8, 4.3-10.5)

6-10 yrs	356 (41.5)	17 (4.4, 2.8-6.7)	65 (18.2, 14.5-22.5)	9 (2.6, 1.5-4.6)

> 10 yrs	19 (2.2)	3 (13.2, -0.2-5.1))	4 (19.7, 8.5-39.4))	2 (13.2, 4.5-32.5)

*p-value*		0.101	0.033	0.015

**Crash status**				

No crash in last five years	765 (89.3)	42 (5.5, 4-7.4)	168 (22, 18.5-25.9)	31 (4.1, 2.8-6)

Crash in last five years	92 (10.7)	12 (12.2, 6.8- 21)	28 (30.1, 23.4-37.7)	8 (8.8, 4.7-15.8)

*p-value*		0.002	0.032	0.003

Income in Pak Rupees (PKR)*				

< 8000	434 (50.6)	24 (5.6, 3.4-9)	99 (22.8, 18.4-27.8)	19 (4.3, 2.4-7.7)

8000-20000	364 (42.5)	28 (7.6, 5.5-10.4)	82 (22.6, 18.3-27.5)	19 (5.3, 3.6-7.9)

> 20000	59 (6.9)	2 (6.3, 0.4-9.4)	15 (25, 15.3-38)	1 (2.1, 0.4-9.4)

*p-value*		0.138	0.92	0.431

*1 USD ≈ 87 PKR

Marijuana usage rate was almost 23% for the driver sample (Table 1). Again this was significantly higher in truckers (30%) compared to bus drivers (14.7%) (Table 2). No significant difference in usage was seen between the different age groups in using marijuana while driving. Sindhis had the highest usage rate (52%) but the second highest usage was in Kashmiris (32%). Education did have an effect on marijuana use with the lesser educated drivers showing higher rates of marijuana usage. Marijuana was used by 30% of the drivers involved in a road crash in the last five years compared to 22% in those without a history of crashes. Alcohol and marijuana were used in conjunction by 4.6% of the sample. Drivers who drove trucks, had Sindhi ethnicity, had more than 10 yrs of education or were over 50 yrs old had the highest conjunction usage rates in the sample. Almost 8% of the drivers also reported using stimulant pills while driving on the road.

In addition to this, the drivers were asked their opinions on why commercial drivers resort to alcohol and marijuana use while driving on long routes (Table 3). Major response categories included usage of alcohol and marijuana for relieving fatigue, sleepiness, mental peace and calm and of course dependence.

**Table 3:** Responses of drivers when asked about Alcohol and Marijuana use among commercial drivers

	Percentage of Responses
**Reasons for Marijuana use**	

1. To get peace and calm	15.3%

2. Drivers feel sleepy without it	12.9%

3. Addiction Dependence	10.2%

4. Relieves fatigue	9.5%

5. Like to smoke marijuana	7.2%

6. Difficult to drive on long routes without it Get pleasure while driving	5.7%

7. Irresponsible behavior	5.3%

8. Did not respond	13.2%

9. Do not know	20.7%

**Reasons for Alcohol use**	

1. Relieves fatigue	17.8%

2. Just like to drink it Habitual drinking	17.0%

3. Irresponsible behavior	9.8%

4. Drivers do not drink alcohol	9.1%

5. Mental peace and calm	7.6%

6. Feel relaxed and driving is easier	6.5%

7. To be able to drive the vehicle faster	5.1%

8. To stay awake while driving	1.2%

9. Do not know	26.9%

We used regression analysis to elucidate potential predictors of alcohol and marijuana use while driving (Table 4). Besides previously mentioned factors, we found higher risks of alcohol use in drivers who had been divorced or widowers compared to those who were married or never married. Marijuana use risk was significantly higher in drivers who reported they were stressed while driving. Road rage behaviours such as fighting with people on the road and racing with other drivers were predictors of both alcohol and marijuana use. Drivers who reported making mistakes while driving such as speed miscalculation of other vehicles were also at a higher risk.

**Table 4:** Association of alcohol and marijuana use with different driver characteristics (regression analysis)

	Alcohol Use	Marijuana Use
	**Unadjusted OR (95% CI)**	**Unadjusted OR (95% CI)**

**Type of respondent**	***< 0.001***	***< 0.001***

Truck driver	5.2 (2.5-10.5)	2.4 (1.7-3.4)

Bus driver	(ref)	(ref)

**Ethnicity**	***0.013***	***< 0.001***

Punjabi	(ref)	(ref)

Sindhi	*5.2 (2-13.3)*	*5.8 (3.2-10.4)*

Pathan	2.5 (1.2-5.3)	1.3 (0.82-1.9)

Kashmiri	1.6 (0.6-4.7)	2.3 (1.4-3.7

Others	2.4 (0.8-6.9)	1.4 (0.73-2.6)

**Marital Status**	***0.038***	*0.342*

Unmarried	(ref)	(ref)

Married	0.96 (0.43-2.1)	0.86 (0.57-1.3)

Divorced	4.3 (1.01-18.3)	1.5 (0.5-4.6)

Widower	5.6 (1.3-24.2)	2.1 (0.6-6.8)

**Stressed while driving**	*0.259*	***< 0.001***

Yes	1.5 (0.75-2.9)	2.6 (1.7-3.9)

No	(ref)	(ref)

**Fight on the road**	***< 0.001***	***< 0.001***

Yes	*3.3 (1.5-7.5)*	*3 (2-4.4)*

No	(ref)	(ref)

**Racing with other vehicles**	***0.002***	***< 0.001***

Yes	*2.9 (1.4-5.9)*	*3.2 (2.2-4.7)*

No	(ref)	(ref)

**Speed miscalculation while overtaking**	***0.001***	***< 0.001***

Yes	2.9 (1.5-5.6)	2.1 (1.5-2.9)

No	(ref)	(ref)

## Discussion

Our results show that DWI, marijuana along with stimulant pill use is a major issue in the commercial driver population of Pakistan. Currently, owing to the fact that sale and use of alcohol is illegal in the Muslim dominant Pakistani population, alcohol use while driving is not considered a major risk factor for road traffic injuries. This common belief is contradictory to our study findings which show up to 10% of truck drivers involved in alcohol use while driving. Our estimates are based on self reports, and as the practice of alcohol use is illegal and frowned upon in general society, we can safely assume that our findings are underestimated and the actual problem is of a greater magnitude. Our results show higher use of alcohol and marijuana in truck drivers compared to bus drivers. A possible explanation for this could be the conditions in which these two different groups drive. Bus drivers transport passengers in their vehicles resulting in there being less of a chance for the driver to engage in such activities without being reprimanded by the passengers.

Prevalence of DWI and marijuana use among commercial drivers is high in Pakistan compared to other countries. Studies on commercial drivers also show lower estimates compared to our study (0.5%-4.9%) [20,29]. This difference may be due to lapses in the enforcement of drug and alcohol use laws by the Pakistani highway police. Some western studies also show high prevalence of substance abuse but these findings are likely to be over-estimated as they have been done in drivers suspected and then checked for substance abuse by the traffic police [30,31].

The drivers' opinions on why alcohol and marijuana is used indicate a lack of awareness of the hazards associated with this practice. Most drivers believed that substance abuse relieves fatigue, makes the journey easier, and even prevents sleepiness, although sleep debt accumulates and cannot be relieved without normal restorative sleep [32]. This presents us with an avenue for intervention to reduce substance abuse in this population. Awareness campaigns for behaviour modification are known to be effective, especially when coupled with promotion of alternative behaviours [33]. The success of these campaign has been greater when they are combined with proper enforcement of stringent laws on drunk driving and substance abuse, and improved awareness of these laws and consequences of violations [33,34].

Laws against DWI are ineffective in the country owing to the lack of use of methods such as random breath analyzer tests and blood tests on drivers suspected of DWI [2]. Evidence of reduced fitness to drive as a consequence of drug consumption is essential for proper law enforcement [35]. Pakistan still does not have a law specifying the allowable blood alcohol concentration of drivers on the road. Designating a BAC level, random checks and breathalyzer tests on these drivers on the road, combined with appropriate awareness campaigns may prove to be effective to resolve this dilemma [17,33]. It is also important to note that regulations need to be enforced not just on the roads but also in the commercial transport organizations which hire the drivers to transport goods or passengers. Regular checks of drug and alcohol should be carried out in all drivers working for such companies for continued employment and the results should be available to law enforcement authorities.

Our results show significant variations in substance abuse among drivers of different ethnicities and socioeconomic strata. These findings indicate psychosocial elements influencing the use of drugs and alcohol in this population which were not explored in our study. Further investigations looking into this aspect may clarify the relationships between social, cultural and psychological risk factors for DWI and provide critical information for targeted and appropriate interventions.

The commercial drivers of large vehicles are a mobile population, spending most of their time on the road and stopping at segregation points from time to time. Our study was done at one of the biggest bus/truck stations in the country, lying on the Karachi-khyber route which runs across the country. The drivers stopping at this point are representative of the Pakistani large vehicle commercial driver population. Furthermore, we used time location cluster sampling, a type of probability sampling for mobile populations, minimizing any selection bias in our results. Our study included general commercial drivers of large vehicles driving on highways, giving us the prevalence estimates for this population and not only of those involved in crashes. This information is not frequently seen in the scientific literature. A limitation of the study is that we did not assess blood alcohol levels and instead resorted to self reporting of the practice by the drivers. There is a high level of agreement between reported and observed information on crashes and RTIS [36] seen but reported information on illegal or socially unaccepted activities such as substance abuse while driving is generally under-reported [37-39]. Considering our objectives and the fact that alcohol use is illegal by law, and disliked in the Muslim dominant Pakistanis society, we believe that self reported alcohol use while driving is a reasonable indicator for the presence of DWI and marijuana use in this population. It can be inferred that the actual problem may be greater than what our study recorded.

## Conclusion

The first step in controlling this situation is recognizing that the problem actually exists. We need to be open about the fact that, despite laws banning alcohol consumption in Pakistan, it is still being used by the driver population of the country. Once this hurdle is crossed, we need to implement more focused and effective interventions controlling alcohol use while driving, along with studies objectively measuring their effect.

## Competing interests

The authors declare that they have no competing interests.

## Authors' contributions

MUM conceived the study, designed and carried it out, and drafted the manuscript. IK assisted in the data collection phase, in the literature review and contributed to the methods section of the final draft. BA and JAR were involved in the design of the study, statistical analysis and contributed to the final draft. All authors read and approved the final manuscript.

## Pre-publication history

The pre-publication history for this paper can be accessed here:

http://www.biomedcentral.com/1471-2458/12/145/prepub
